# In vivo closed-loop control of a locust’s leg using nerve stimulation

**DOI:** 10.1038/s41598-022-13679-z

**Published:** 2022-06-27

**Authors:** Francisco Zurita, Fulvia Del Duca, Tetsuhiko Teshima, Lukas Hiendlmeier, Michael Gebhardt, Harald Luksch, Bernhard Wolfrum

**Affiliations:** 1 Neuroelectronics, Munich Institute of Biomedical Engineering, Department of Electrical and Computer Engineering, Technical University of Munich, 85748 Garching bei München, Germany; 2 Medical and Health Informatics Laboratories, NTT Research Incorporated, 940 Stewart Drive, Sunnyvale, 94085 CA USA; 3 Chair of Zoology, Technical University of Munich, 85354 Weihenstephan, Germany

**Keywords:** Motor control, Peripheral nervous system, Soft materials, Electrical and electronic engineering

## Abstract

Activity of an innervated tissue can be modulated based on an acquired biomarker through feedback loops. How to convert this biomarker into a meaningful stimulation pattern is still a topic of intensive research. In this article, we present a simple closed-loop mechanism to control the mean angle of a locust’s leg in real time by modulating the frequency of the stimulation on its extensor motor nerve. The nerve is interfaced with a custom-designed cuff electrode and the feedback loop is implemented online with a proportional control algorithm, which runs solely on a microcontroller without the need of an external computer. The results show that the system can be controlled with a single-input, single-output feedback loop. The model described in this article can serve as a primer for young researchers to learn about neural control in biological systems before applying these concepts in advanced systems. We expect that the approach can be advanced to achieve control over more complex movements by increasing the number of recorded biomarkers and selective stimulation units.

## Introduction

Interfacing the nervous system with stimulation and recording devices can be used to diagnose and treat nervous system disorders. Such neurotechnology-based concepts are successfully exploited in a variety of medical applications: in the central nervous system, cochlear implants are routinely used to restore hearing by directly stimulating the auditory nerve^1^. In addition, deep brain stimulation is applied to treat symptoms from Parkinson’s disease and depression^2,3^. Furthermore, several strategies for specific disease treatment have recently evolved based on bioelectronic interfacing with the peripheral nervous system. For example, electric stimulation of the vagus nerve has been used for the treatment of epilepsy, and depression, and research is being conducted on its effectiveness to treat heart conditions^4–7^. Stimulation of the posterior tibial nerve has been investigated to treat incontinence^8^ and transcutaneous electrical nerve stimulation (TENS) has long been used as a palliative for chronic pain^9^. Applying neural modulation in humans is difficult due to complex, time-dependent, and largely uncharacterized response behavior. This means that the same stimulus may yield different responses, not only when applied in different subjects (inter-subject variability)^10^, but also within the same subject at different times (intra-subject variability)^11,12^. Since the most commonly applied method of modulating neural activity is in an open-loop fashion, i.e., without monitoring the output variable, the response has high variability. To account for this variability, a closed-loop strategy is crucial to monitor the desired outputs and adapt the modulation parameters accordingly to achieve some degree of control over the output variable^13–18^. The concept of control in the scope of this article should be understood from a control theory perspective, in which the use of an output signal of a system is used to modify the command signal of this system to reduce the difference between the desired and actual output signal^19^. Sun et al. review four clinical cases where closed-loop systems were used with better results compared to open-loop systems^20^. Kassiri et al. discuss the benefits of closed-loop stimulation in the treatment of epilepsy^21^. In view of the advantages, new devices with potential for closed-loop applications are emerging for the peripheral^22,23^, and the central nervous system^24,25^. In particular, implantable and potentially adaptive systems are desired for long-term studies. In this context, multisite electrodes have been leveraged to selectively stimulate appropriate nerve fibers^26^ and miniaturization as well as power delivery strategies have been implemented^27,28^.

Fundamental research on closed-loop bioelectronic intervention strategies is typically carried out using animal models, usually rodents and other smaller mammals. For example, closed-loop vagus nerve stimulation has been shown to enhance recovery of spinal cord injury in rats^29^, and a closed-loop gait control system has been demonstrated in a cat^30^. Furthermore, a closed loop approach targeting the peripheral nervous system has been shown for bladder control using optogenetic methods in mice^31^. Different strategies have also been explored to achieve a closed-loop control using frequency-modulated stimulation. Wenger et al. presented a neuromodulation approach for gradual control of mammalian gait, by targeting the spinal cord and recruiting the sensory fibers that elicit a leg reflex movement^32^. Choosing an appropriate control approach can enhance fiber selectivity, which is important to prevent eliciting undesired responses^33^.

As an alternative to mammals, insects prove to be useful animal models for fundamental research as well as educational purposes. Their nervous system is simpler than that of mammals and allows for more precise targeting of the tissue. Additionally, in vitro studies with insects permit the analysis and characterization of their different subsystems, isolated from the influence of the rest of the body^34^. Insects have also inspired computational models to test various control strategies, making them a versatile model^35–37^. While different approaches and electrode designs have been proposed to interface with peripheral nerves in literature^38,39^, the small size of peripheral nerves limits their applicability in insects. For this reason, many in vivo experiments carried out on insects attempt to close the loop by directly applying electrical stimulation to the target muscles instead of the nerve innervating them^40–42^.

In this work, we demonstrate the control of a locust’s leg angle extension via a feedback-loop strategy by directly stimulating one of the extensor motor nerves. A nerve cuff is fabricated using a 3D printed mold allowing rapid prototyping of the interface. The elicited leg movement is measured with a commercial flexible resistor and controlled in real time using a train of digital-level voltage pulses implemented with a microcontroller.

## Materials and methods

### Control loop design

**Figure 1:**
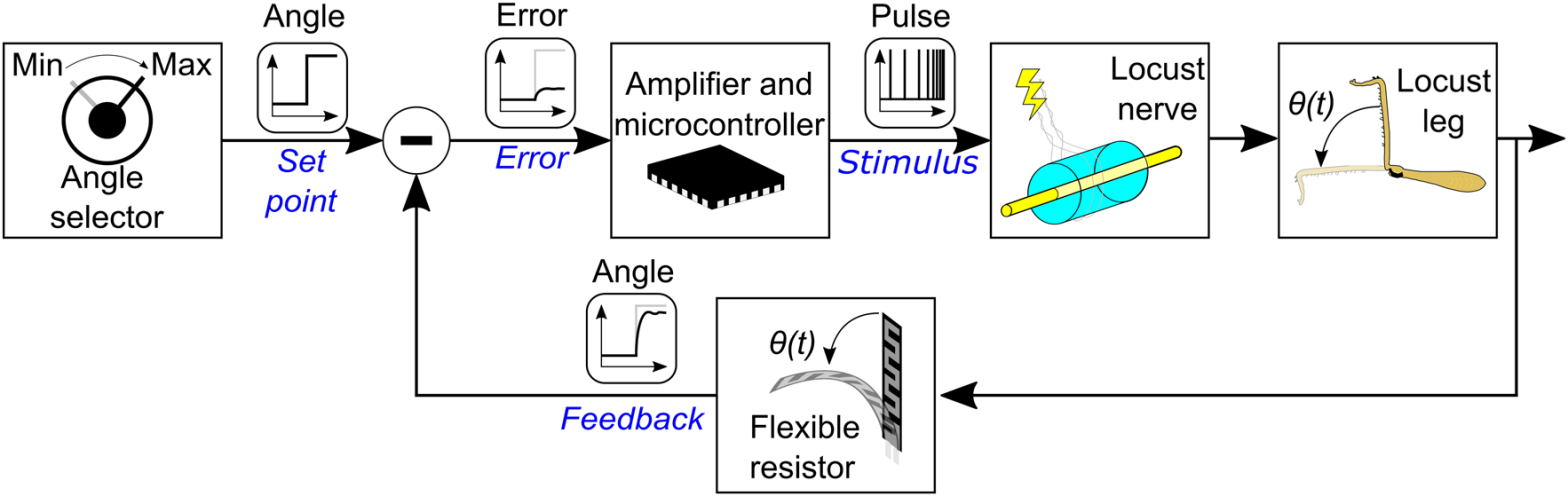
Scheme of the implemented control loop. The angle set point is selected and the measured leg angle subtracted from it to conform the error signal, which is sampled by the microcontroller. A proportional control algorithm runs in the microcontroller, varying the period of the stimulus signal applied on the nerve. The leg angle is sensed with a flexible resistor to provide the feedback.

Figure 1 shows a scheme of the proposed control loop. It starts with the selection of the desired angle set point. The feedback signal is then subtracted from the set point signal to produce an error signal. We implemented the subtraction with an operational amplifier (TL094, Texas Instruments, Dallas, USA) in a differential configuration. Both signals are buffered with operational amplifiers in voltage-follower configurations before subtraction and the amplitude of the feedback signal is calibrated to match the amplitude of the set point signal. The error signal is sampled using the internal analog-to-digital converter (ADC) of the microcontroller (nRF52840, Nordic Semiconductor, Oslo, Norway) at a sampling rate of 10 ksps. We configured the microcontroller to generate a train of monophasic pulses with a fixed pulse width of 100 μs and an amplitude of 3 V. The period of the applied train pulses is modulated proportionally to the error signal. We limited the duration of the pulses to 100 μs to avoid redox reactions that could be generated due to the large amplitude of the stimulation. The period of the output is calculated as $T = 250 {\text{ms}} - A \cdot e\left(t\right),$ where $T$ is the output period, $e\left(t\right)$ is the error signal in V, and $A$ is an amplification factor, which was empirically set to 0.36 s V^−1^. The output period is clipped to a minimum of 50 ms and a maximum of 250 ms. A flexible resistor (ZD10-100, Fafeicy) attached to the leg using a 50-μm nylon string measured the joint angle. A set of pulleys guide the string along a path that increases the bending of the resistor with the joint angle and thus, increases the error signal. The angle span goes from 0° to 90°, making the flexible resistor change its impedance from 50 to 3 kΩ.

### Electrode fabrication

**Figure 2:**
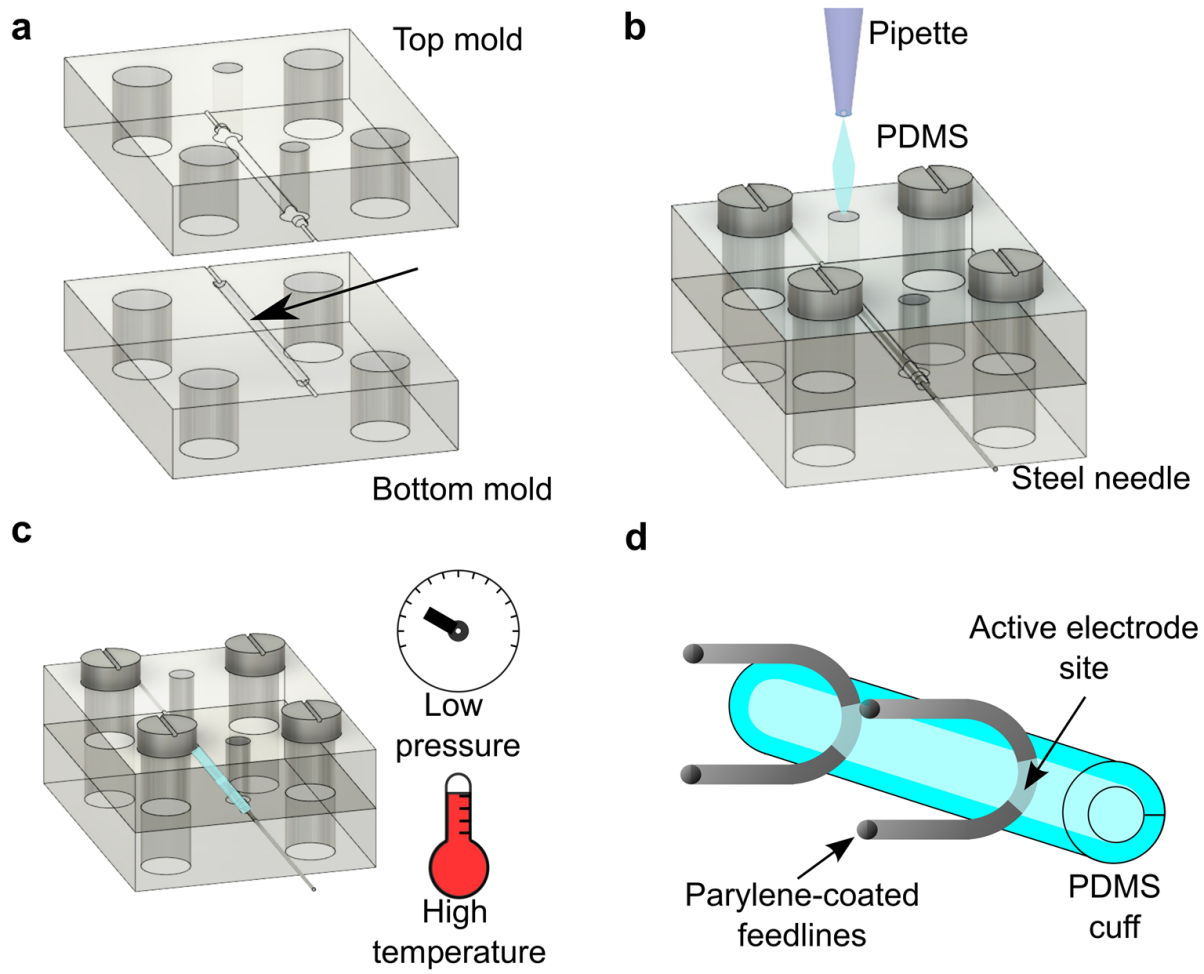
PDMS cuff fabrication steps. (**a**) The mold for the cuff is 3D printed as two separated parts. After assembly, the two parts form a cavity with the desired dimension of the cuff. (**b**) The molds are tightly screwed together, and a needle is placed inside the mold to form the cuff’s lumen. PDMS is cast inside the resulting tube-shaped cavity. (**c**) The excess air in the PDMS is removed by degassing in a vacuum chamber and the PDMS is thermally cured. (**d**) After curing, the tube is detached from the mold and the stimulation electrodes are inserted through the PDMS tube. The cuff is then cut to a length of ~ 1 mm and opened along the tube to enable nerve insertion. Finally, the exterior of the electrode is coated with parylene-C.

We implemented a simple 3D fabrication approach that allows rapid prototyping of cuff electrodes. The cuff electrodes were designed to fit the diameter of the targeted nerve. First, we fabricated molds to shape the cuff into a cylinder. The layout for the molds was designed using a 3D computer-aided design software (Fusion 360, Autodesk, Inc., San Rafael, USA). We fabricated the molds with a commercial resin (Detax Medicalprint Clear, 385 nm, Detax GmbH & Co. KG, Ettlingen, Germany) using a stereolithographic 3D printer (MiiCraft 50X, MiiCraft, Hsinchu, Taiwan). The mold was separated into top and bottom parts with a cylindrical cavity of 900 μm in diameter to cast the substrate of the cuff. Two orifices were included in the top part of the mold for insertion of the cuff material and venting the air trapped inside the cavity during the pipetting process (Fig. 2). The molds further included supporting structures to align a steel needle of 150 μm in diameter along the cylindrical cavity, which formed the lumen of the cuff. After the mold was assembled and the top and bottom parts were screwed together, the cuff material was pipetted into the cavity with the needle in its center (Fig. 2b). Silicone elastomer, polydimethylsiloxane (PDMS, Sylgard 184, Dow Chemical, Midland, USA) at a 5:1 base-to-catalyzer ratio was used as cuff material to provide sufficient stiffness for withstanding the latter insertion procedure with the nerve. We placed the mold with the uncured PDMS into a low-pressure chamber overnight to remove the trapped air and avoid the formation of bubbles in the PDMS. Subsequently, we cured the PDMS in an oven at 80 °C for around 8 h (Fig. 2c). After curing, we detached the PDMS tube from the mold and removed the needle. Two silver wires (127 μm in diameter) were inserted through the tube so that the wire would only tangentially touch the inner wall of the tube (Fig. 2d). The wires were separated by ~ 500 μm from each other. Finally, the cuff was cut to a length of 1 mm and opened along the side of the tube to provide access for the nerve insertion. We coated the electrode with a 3 μm-thick layer of parylene-C to passivate the exposed wire outside of the cuff using a chemical vapor deposition system (Plasma Parylene Systems GmbH, Rosenheim, Germany). Inside the deposition system, dichloro-di(p-xylylene) (Daisan Kasei, Japan) was first vaporized at 150 °C and then pyrolyzed at 740 °C to generate chloro-p-xylylene monomer. The monomer was then deposited at room temperature. Finally, we manually removed any traces of parylene insulation from the active electrode sites inside the cuff.

### Electrochemical characterization of the electrodes

We performed chronoamperometry (CA) and impedance spectroscopy (EIS) to characterize the electrodes inside the nerve cuff using a two-electrode configuration vs. open circuit potential (OCP) (PalmSens4, Palmsens, Houten, The Netherlands). Both measurements were done in locust’s saline solution (147 mM NaCl, 10 mM KCl, 4 mM CaCl_2_, 3 mM NaOH and 10 mM HEPES buffer, Sigma Aldrich, St. Louis, USA)^43^. One electrode was set as the working electrode and the other was set as the combined reference and counter electrode. Voltage steps of 50 mV, 1 V and 3 V were applied in the CA measurement, after a stabilization period of 1 s to OCP. The EIS measurement was performed between 5 Hz and 500 kHz, with a sinusoidal signal of 10 mV of amplitude. Cyclic voltammetry scans were applied between − 1 and + 1 V at 10 mV s^−1^ for 10 cycles to precondition the surface of the electrodes before each CA and EIS measurement.

### Surgery and nerve interfacing

**Figure 3:**
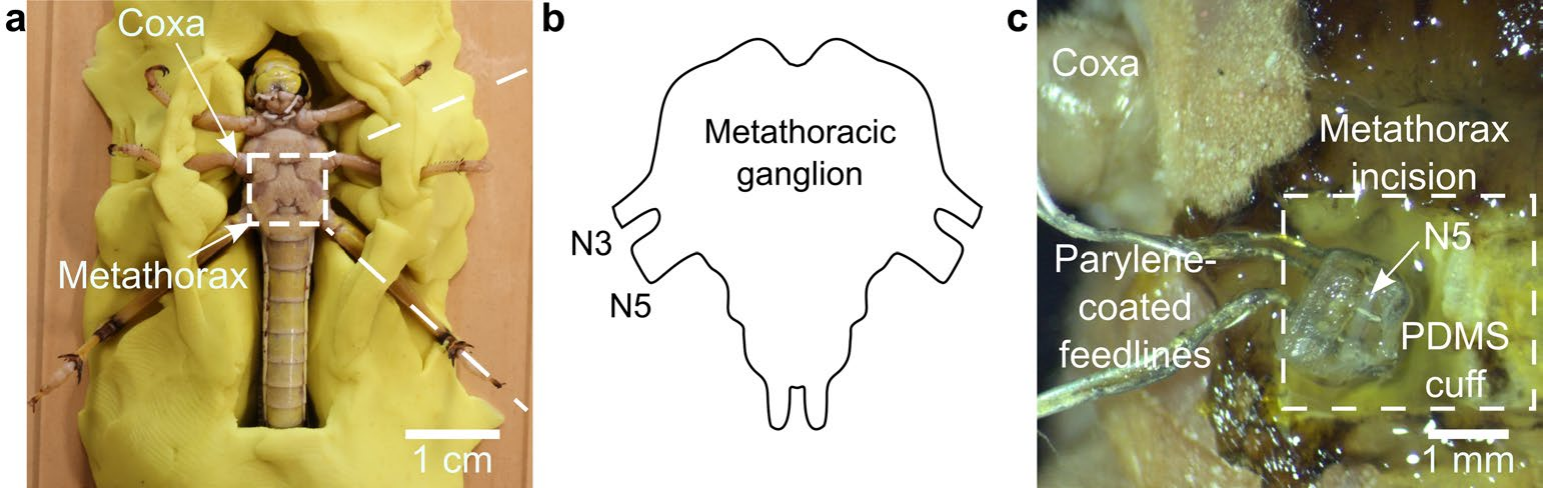
(**a**) Locusta migratoria prior to the surgery in clay bed. (**b**) Scheme of the metathoracic ganglion and the nerves innervating the hind leg. (**c**) Microscope image of the surgical incision on the metathorax of the locust, showcasing the N5 immediately before insertion in the cuff electrode.

We chose the insect *Locusta migratoria* for the experiments because it has a relatively simple nervous system and opted for the tibiofemoral angle of the hind leg as biomarker. The experimental unit consisted of an adult female. We controlled the angle by stimulating the fast extensor tibiae motoneuron (FETi). This neuron has its soma and dendritic branches in the metathoracic ganglion and projects its axon into the nerve 5 (N5) to innervate the ETi, as shown in Fig. 3. Prior to the surgery, we anesthetized the locust by chilling it to 2 °C for around 30 min. Then, we placed it in a holder made of modelling clay with the ventral side facing upward, as shown in Fig. 3a. For the surgery, we removed the cuticle of the metathorax and carefully cut the muscles attached to it, to expose the metathoracic ganglion. Next, we removed the air sacs and the trachea to reveal the desired nerve, which innervates the metathoracic legs. With the aid of a micromanipulator, we slid N5 into the cuff electrode (Fig. 3c). The electrodes were used to stimulate the FETi by delivering the appropriate pulses at a frequency determined by the controller. Finally, we connected the electronic setup and recorded the set point, the error signal, and the pulse train using an oscilloscope (InfiniiVision DSOX2024A, Keysight, Santa Rosa, USA). As angle set points we applied step and ramp functions to characterize the response of the locust movement to different stimuli conditions. In addition, we captured the displacement of the leg on video and extracted the extension angle using image processing (MATLAB R2020b, Mathworks). To this end, the algorithm determined the geometric center of the leg for each frame and calculated the angle via the difference between the center points of each frame and the center point of the first frame, with the tibiofemoral joint as origin.

## Results and discussion

### Electrode characterization

**Figure 4:**
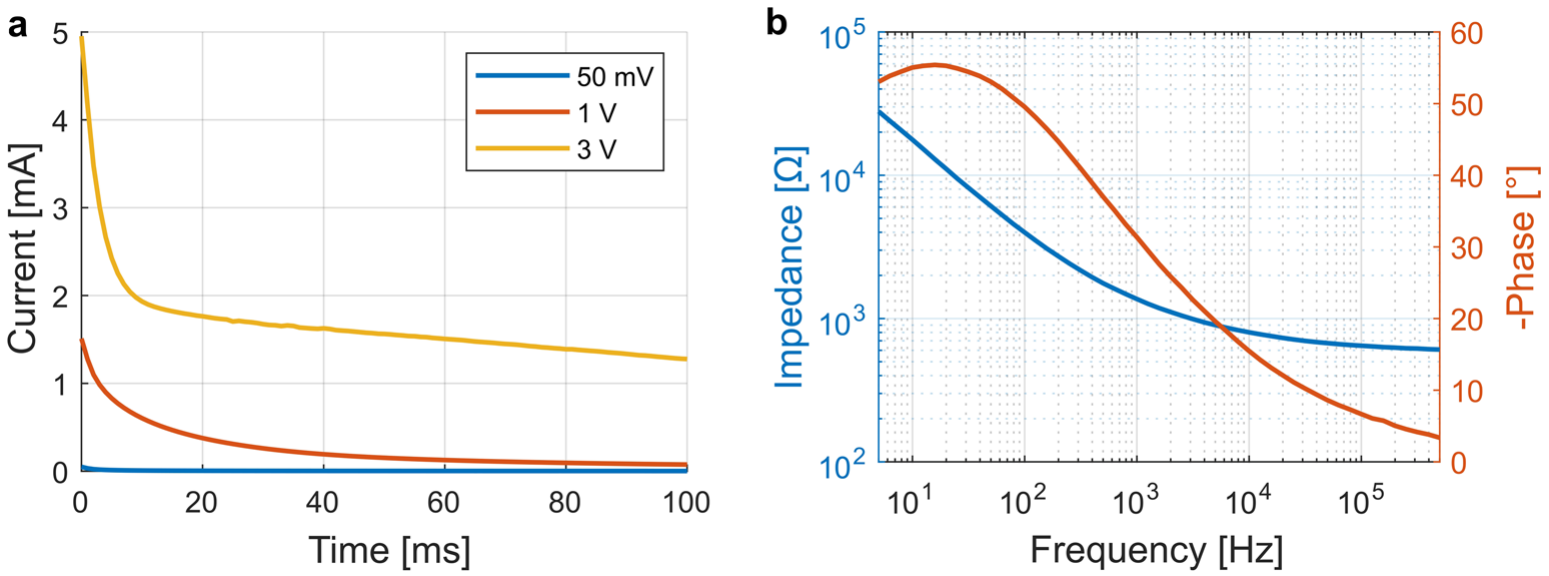
Electrochemical characterization of the custom-made cuff electrodes in saline solution. The characterization was performed in a bipolar configuration between the two stimulation electrodes in the cuff. (**a**) Chronoamperogram for three different voltage step amplitudes. (**b**) Impedance spectroscopy analysis exhibiting a combined impedance of ~ 1.4 kΩ @ 1 kHz.

We characterized the electrodes before implantation using chronoamperometry and impedance spectroscopy (Fig. 4). Figure 4a shows the results of chronoamperometry applying voltage steps ranging from 50 mV to 3 V. As expected, the current response scales with the amplitude. For a voltage step of 50 mV we observe a capacitive spike in the range of tenths of μA. Afterwards, the current quickly decays to 0 μA due to the negligible impact of Faradaic contributions at such a low voltage. For higher voltages we observe a more pronounced contribution of the Faradaic current. At a voltage step of 3 V, the capacitively induced exponential current decay is only dominant during the first 10 ms. Afterwards we observe a slower decay, which is likely caused by effects of diffusive mass transport limiting the Faradaic current. To minimize possible aversive effects due to the presence of Faradaic reactions, we fixed the pulse width to 100 μs, where the capacitive contribution to the current is dominant. Reducing Faradaic reaction in in vivo implementations is important, as both DC currents and the species produced electrochemically at the electrode can damage the nerve tissue^44^. The EIS results are shown in Fig. 4b, showcasing an absolute impedance of both electrode–electrolyte interfaces in series around 1.4 kΩ at 1 kHz.

### Control strategy

**Figure 5:**
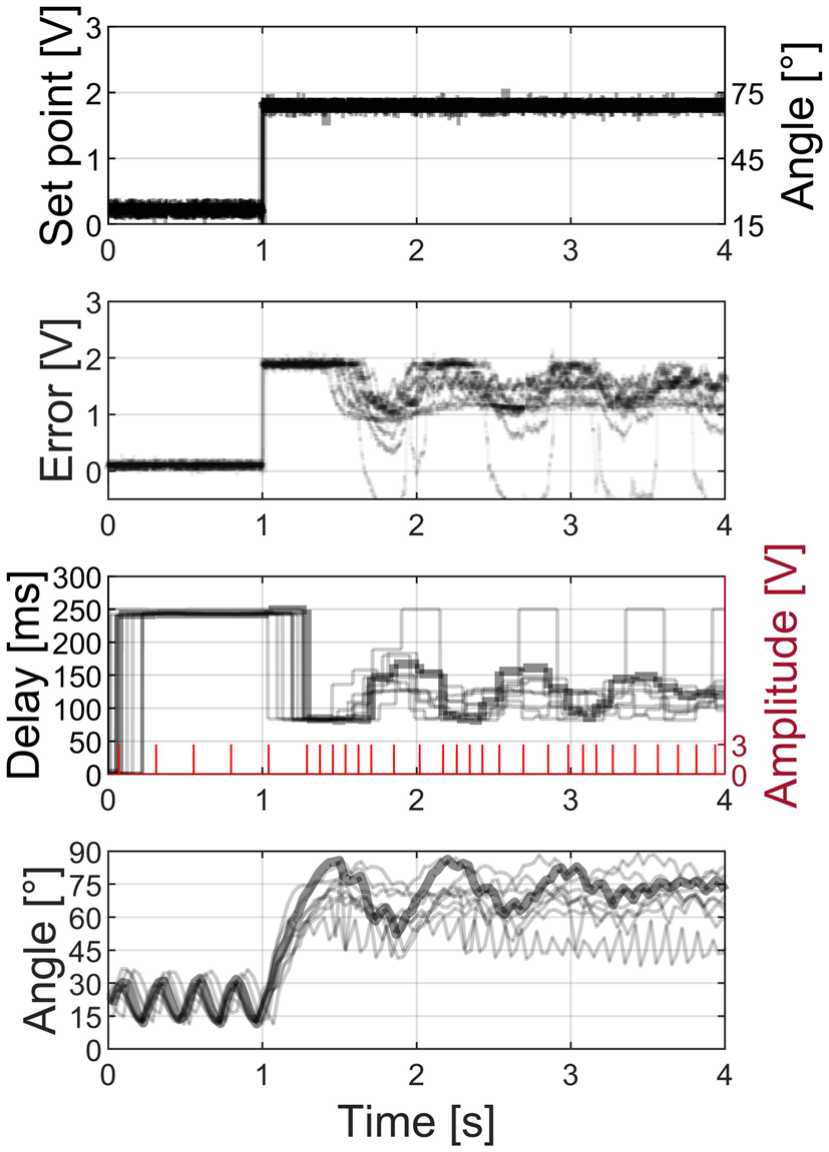
A set point voltage is applied to determine the desired angle of the leg. The error between set point and feedback signals is converted into a stimulation pulse pattern (red), and a corresponding inter-pulse delay. The stimulation pattern triggers a response in the leg angle, which is extracted from video recordings. The black traces show 10 repetitions of the same experiment with the thicker black traces corresponding to the indicated pulse pattern.

Figure 5 shows the results for the 10 trials of the voltage step stimulation. The set point signal was given by a ~ 2 V step function. As a guiding value, we related this voltage level to an angle of 75°, estimated from the mean angle in the experimental results. The change in the set point produced an error signal that was encoded into a stimulation rate via the microcontroller. The limb reacted to the change of stimulation rate and produced an extension of the locust’s leg captured by video recordings. The mechanical response was additionally sensed by the flexible resistor, increasing the feedback and decreasing the error signal. As expected, this led to an initial error maximum, due to the inertia of the leg movement. This abrupt transient can be mitigated by the incorporation of a derivative factor in the control loop. Additionally, the steady-state error can be eliminated by the incorporation of an integrative factor. The delay between successive stimulation pulses is maximum when the error signal is minimum and decreases abruptly at the step onset instant.

**Figure 6:**
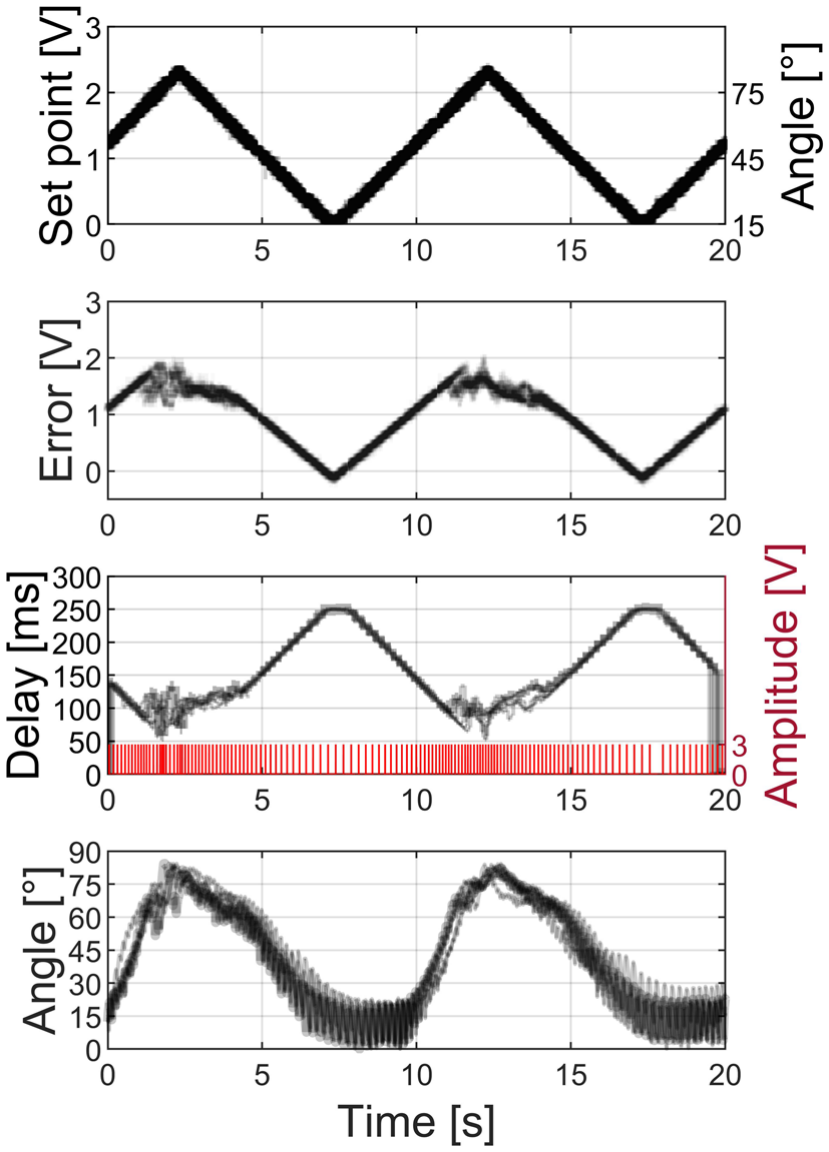
A cyclic voltage ramp set point is applied to generate movement patterns of a locust’s leg. The error signal between set point and feedback signals is converted into a stimulation pulse pattern (red), and a corresponding inter-pulse delay. The stimulation pattern triggers a response in the leg angle, which is extracted from video recordings. The black traces show 5 repetitions of the same experiment with the thicker black traces corresponding to the indicated pulse pattern.

In addition to the step response, we analyzed the closed-loop stimulation of the locust’s leg to a continuously varying control signal in form of a cyclic ramp. The signals for the ramp stimuli are presented in Fig. 6 (shown as 5 traces of 2 ramp cycles). The individual signals were obtained as previously described for the step response. As we can see in Fig. 6, the locust’s leg followed the ramp control signal repetitively. Interestingly, an asymmetry between extension and flexion of the leg was observed in the experiment. The extension followed the control signal with a steeper response. Furthermore, an oscillation was evident at low angles (flexion state). These results can be explained by the chosen configuration for rate-based stimulation of the extensor nerve. At very low frequencies, we observe the response to individual pulses, which deviate the leg from the flexion state, which is not directly controlled by stimulation in our experiments. Furthermore, the flexion of the leg and return to resting position is determined by the tendon of the tibiofemoral joint and the intrinsic neural activity and reflexes of the locust. To achieve more precise control of the leg position, both the flexor and extensor nerve should be controlled independently and the natural feedback mechanisms suppressed.

**Figure 7:**
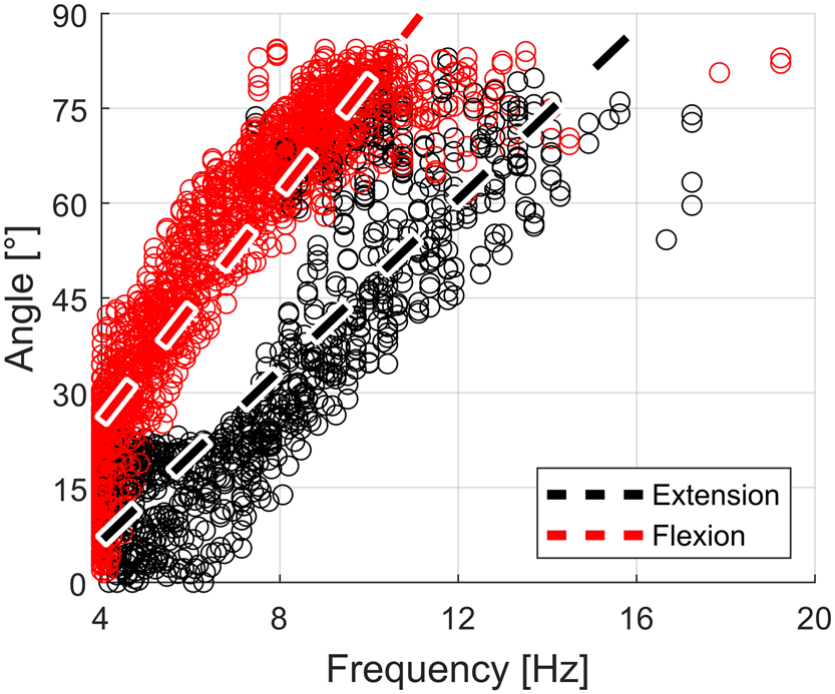
Scatter plot of the angle of the leg versus the frequency of stimulation, computed as the inverse of the inter-pulse delay, for the voltage ramp experiment. The upper cluster (red) represents the trajectory of flexion of the leg, whereas the lower cluster (black) represents the trajectory of extension of the leg. The linear fits are computed using the method of least-squares.

We analyzed the correlation between the frequency (computed as the inverse of the inter-pulse delay) and the angle of the leg for the voltage ramp experiment. Figure 7 shows the scattering of the angles corresponding to different frequencies. Two linear clusters can be identified, one corresponding to the flexion and the other to the extension of the leg. The separation of the same trajectory in two different clusters might be explained by feedback signals on the flexor nerve, which possibly depend on the previous state.

The results demonstrate that it is possible to control the angle of a locust’s hind leg using a simple, closed-loop stimulation strategy. In particular, the control was achieved using short voltage pulses and varying the delay between them. Fast pulse-generation devices can be implemented using simple commercial microcontrollers, which offer configurable outputs for multisite stimulation and are easily accessible. Furthermore, fast pulses are advantageous because they allow a large proportion of the current to be capacitive instead of faradic, thus decreasing the occurrence of redox reactions, which could be harmful to the surrounding tissue^45^.

Multiple aspects could be advanced to improve the precision of this intervention in future implementations. Most importantly, a single stimulation site and a simple proportional control strategy limit the degree of precision that can be achieved. Consequently, multi-site selective stimulation of a single nerve or coordinated stimulation of different nerves are paramount for precise control using this concept^46,47^. Another important factor for reliable control is the transducer. In this work, we opted for an ink-based flexible resistor, because we wanted to minimize the mechanical load on the leg. However, the nonlinear response of the chosen transducer influences the control strategy. Other angle transducers, such as potentiometers, could represent a very high mechanical load for the leg of the locust, thus interfering with the free movement. A low-friction rotary encoder with an analog-voltage output or more advanced thin-film flexible resistors could be the best compromise between precision and mechanical load. Finally, while a proportional control strategy sufficed for our purposes, more complex strategies would bring additional benefits, such as the absolute elimination of the error signal with the addition of an integrative factor in the control loop. The demonstrated concept of controlling a variable in a non-fully characterized system might be applied to other systems such as the degradation of fat into heat by stimulating nerves innervating adipose tissue and monitoring its temperature as control parameter.

## Conclusions

We demonstrated a closed-loop stimulation system to control the angle of extension of a locust’s leg in real time by stimulating its extensor nerve with custom cuff electrodes. The system is implemented by a microcontroller that generates a rate-coded stimulation signal, and a flexible resistor provides the feedback signal. The results show that the average leg angle can be controlled using a time-dependent input and a stimulation signal between approximately 4 and 20 Hz. We expect this model to serve as a primer for students and young researchers interested in applying control theory to biological systems. Future work should address selective stimulation approaches to allow a finer control of leg motion.

## Supplementary Information

Supplementary Information 1.

Supplementary Information 2.

Supplementary Information 3.

Supplementary Video 1.

Supplementary Information 4.

Supplementary Information 5.

Supplementary Video 2.

Supplementary Information 6.

Supplementary Information 7.

Supplementary Information 8.

## Data Availability

The datasets generated during and/or analysed during the current study are available from the corresponding author on reasonable request.
