# Supplementary material for: In vivo closed-loop control of a locust’s leg using nerve stimulation
Source: Sci Rep. 2022 Jun 27;12:10864. doi: 10.1038/s41598-022-13679-z (PMC9237135; doi:10.1038/s41598-022-13679-z)
Supplement: Supplementary file 1 — Supplementary Information 1. [file 41598_2022_13679_MOESM1_ESM.docx]

Supplementary Information

In vivo Closed-loop Control of a Locust’s Leg using Nerve Stimulation

Francisco Zurita^1,2^, Fulvia Del Duca^1,2^, Tetsuhiko Teshima^1,2^, Lukas Hiendlmeier^1,2^, Michael Gebhardt^3^, Harald Luksch^3^ and Bernhard Wolfrum^1,2^

^1^ Neuroelectronics, Munich Institute of Biomedical Engineering, Department of Electrical and Computer Engineering, Technical University of Munich, Garching bei München, 85748, Germany

^2^ Medical & Health Informatics Laboratories, NTT Research Incorporated, East Palo Alto, CA 94303, U.S.A.

^3^ Chair of Zoology, Technical University of Munich, Weihenstephan, 85354, Germany

1. Electrode manufacturing

The following files are provided as supporting material for replication of the cuff electrode fabrication.

Technical drawings:

1. *Tube_mold_bottom.pdf*
2. *Tube_mold_top.pdf*

Design files:

1. *BottomMold.stl*
2. *TopMold.stl*

Instructive video: We include a ~5:20 minute tutorial video on the most important steps to fabricate a silicone cuff electrode. The speech in the video was artificially generated through the website https://ttsmp3.com/.

1. *ElectrodeManufacturing.mp4*

2. Surgical procedure

The surgical procedure is captured in an educational video (~6:30 minutes). The video covers the topics of surgery preparation, surgical procedure, and nerve handling. The speech in the video was artificially generated through the website https://ttsmp3.com/.

Instructive video:

1. *SurgicalProcedure.mp4*

The following procedural aspects help to avoid potential caveats during the procedure:

1. The locust should be fixed immediately after chilling, or it will elicit strong movements if warmed up. The experiment can be conducted at a low temperature for easier handling.
2. The locust should not be maintained in a freezer to ensure vitality.
3. During the surgery, the locust may produce a dark liquid from its mouth. This liquid should be quickly absorbed with tissue, as it can damage the nerves if it gets in contact.
4. The locust should be properly fixed in the modeling clay bed. However, a gap in the clay bed to the sides of the locust at the level of the metathorax should be provided to allow breathing.
5. A superficial cut on the metathoracic cuticle should be performed to avoid damage to trachea and ganglion.
6. The cavity should be kept moist with saline solution to avoid drying of the nerve tissue.
7. The locusts should be handled with care. Excessive stimulation of the nerve (frequency or current) should be avoided. Furthermore, locusts should not be grabbed from the leg. In both cases, the animal will tend to autotomize (detach) the compromised limb.
8. For termination, the locust can be placed in a freezer overnight or dipped in liquid nitrogen for ~10 s.

3. Technical information

A schematic for the sensory and control circuits is provided in Figure S1.


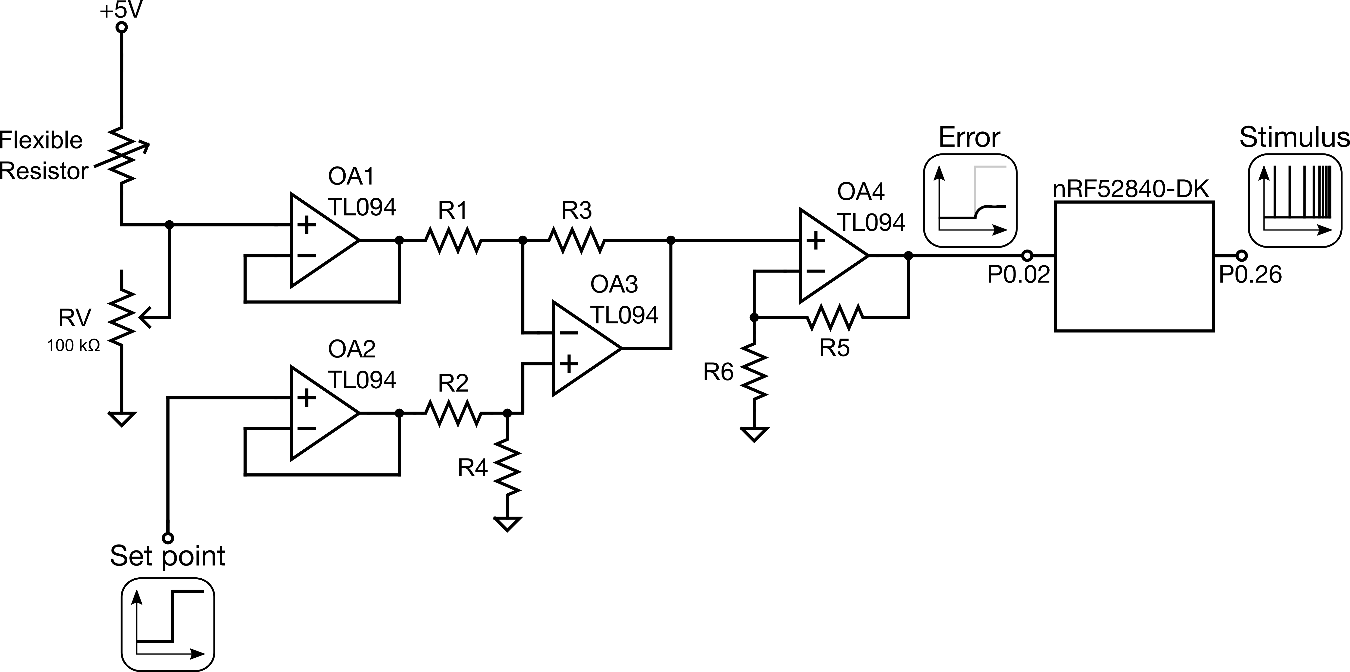


Figure S1 Schematic of the system used to close the control loop.

The components used are a Nordic nRF52840-DK, flexible resistor, 100 kΩ potentiometer, TL094 operational amplifier, resistors to set amplification gain in the differential (R1-R4) and gain (R5, R6) stages.

The values of R1, R2, R3, and R4 set the preamplification of the Set Point and Feedback signals, so the voltage range of the feedback matches the desired set point. The Error signal is then calculated as:

$$Error=Set Point\cdot\left( \frac{R4}{R2+R4} \right)\cdot\left( \frac{R1+R3}{R1} \right)-Feedback\cdot\left( \frac{R3}{R1} \right)$$

R5 and R6 are used to set the amplification gain before signal acquisition by the microcontroller.

The code “*Control_Loop.hex*” from the SI can be directly loaded onto the microcontroller. It is programmed to receive the error signal from P0.02 and generate the pulse signal from P0.26. The output pulse delays are clipped between 50 ms and 250 ms. The potentiometer and non-inverting gain stage can be used to calibrate the desired error signal, e.g., to produce maximum error when the flexible resistor is fully bent, or within a certain bending range. A generic source code (*main.c*) is provided to program the board and tune the digital amplication gain, sampling rate, and limiting frequencies of the stimulation signal to any particular experimental conditions.
