# Supplementary material for: In vivo closed-loop control of a locust’s leg using nerve stimulation
Source: Sci Rep. 2022 Jun 27;12:10864. doi: 10.1038/s41598-022-13679-z (PMC9237135; doi:10.1038/s41598-022-13679-z)
Supplement: Supplementary file 9 — Supplementary Information 7. [file 41598_2022_13679_MOESM9_ESM.pdf]

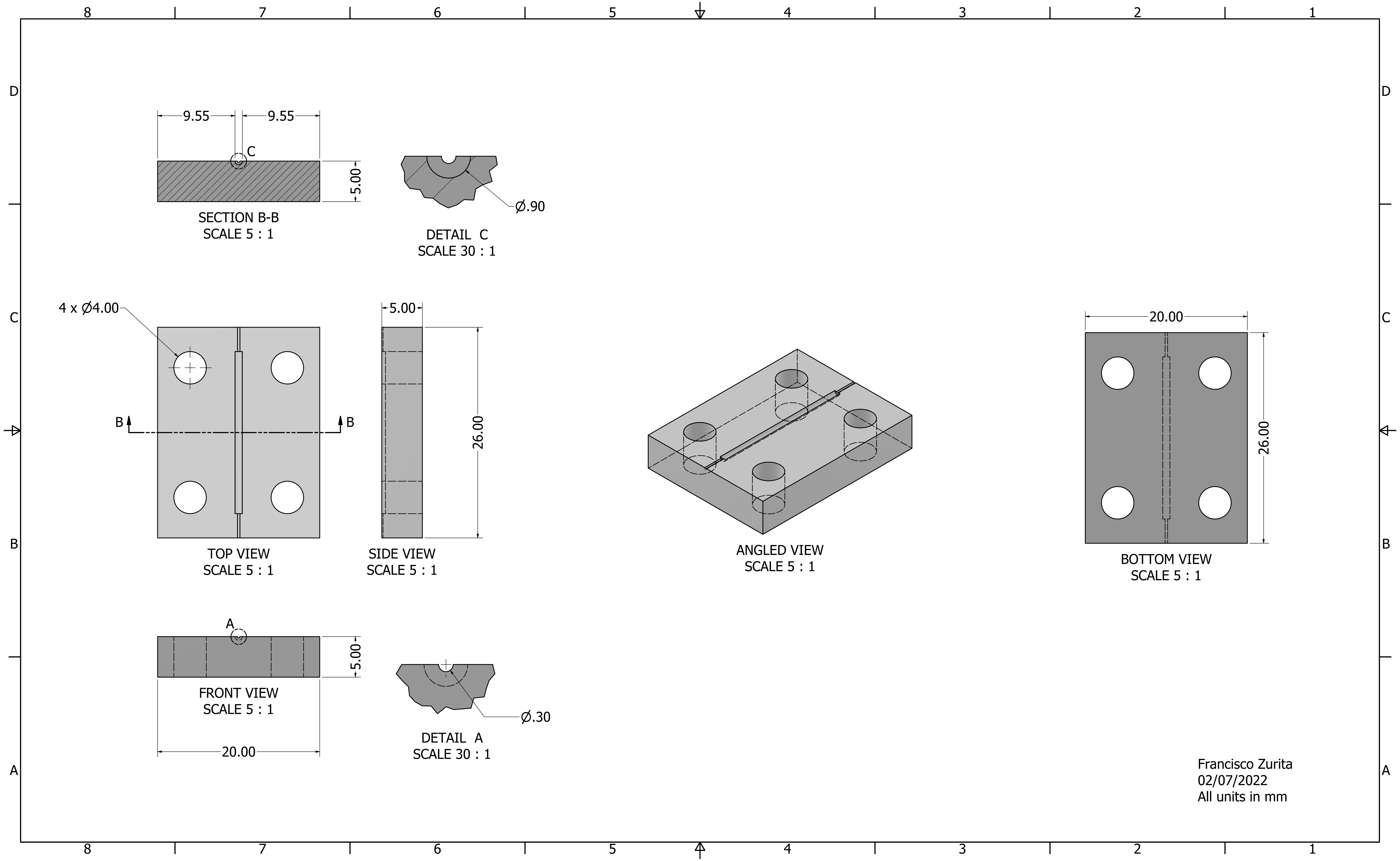
9.55
9.55
C
5.00
SECTION B-B
SCALE 5 : 1
Ø.90
DETAIL C
SCALE 30 : 1
4 x Ø4.00
B
B
TOP VIEW
SCALE 5 : 1
5.00
26.00
SIDE VIEW
SCALE 5 : 1
ANGLED VIEW
SCALE 5 : 1
20.00
26.00
BOTTOM VIEW
SCALE 5 : 1
A
5.00
FRONT VIEW
SCALE 5 : 1
20.00
Ø.30
DETAIL A
SCALE 30 : 1
Francisco Zurita
02/07/2022
All units in mm
