# Supplementary figures and images for: In vivo closed-loop control of a locust’s leg using nerve stimulation
Source: Sci Rep. 2022 Jun 27;12:10864. doi: 10.1038/s41598-022-13679-z (PMC9237135; doi:10.1038/s41598-022-13679-z)

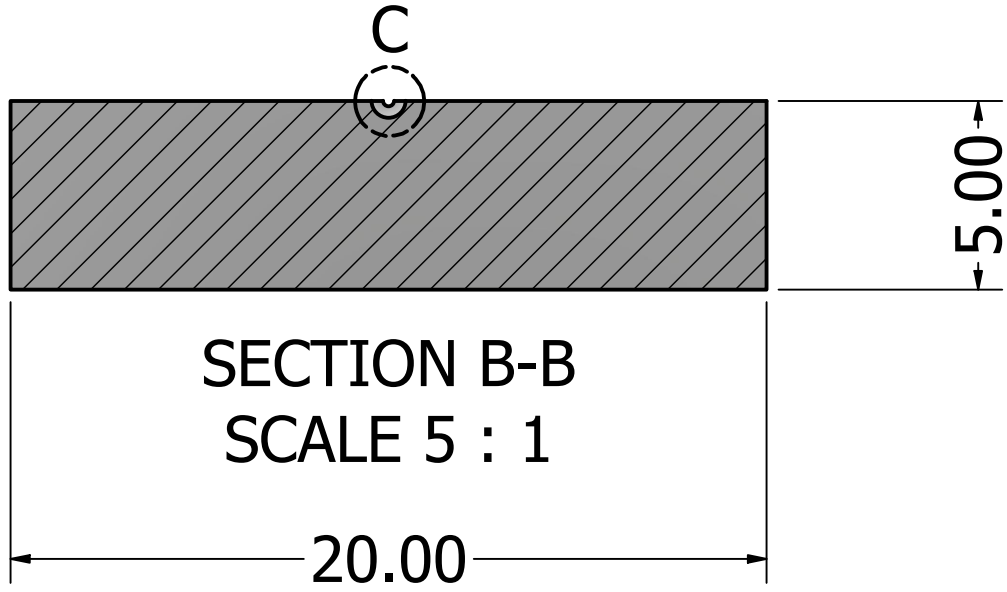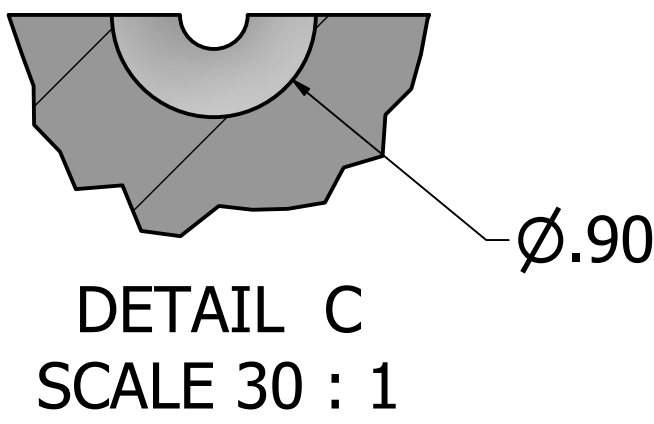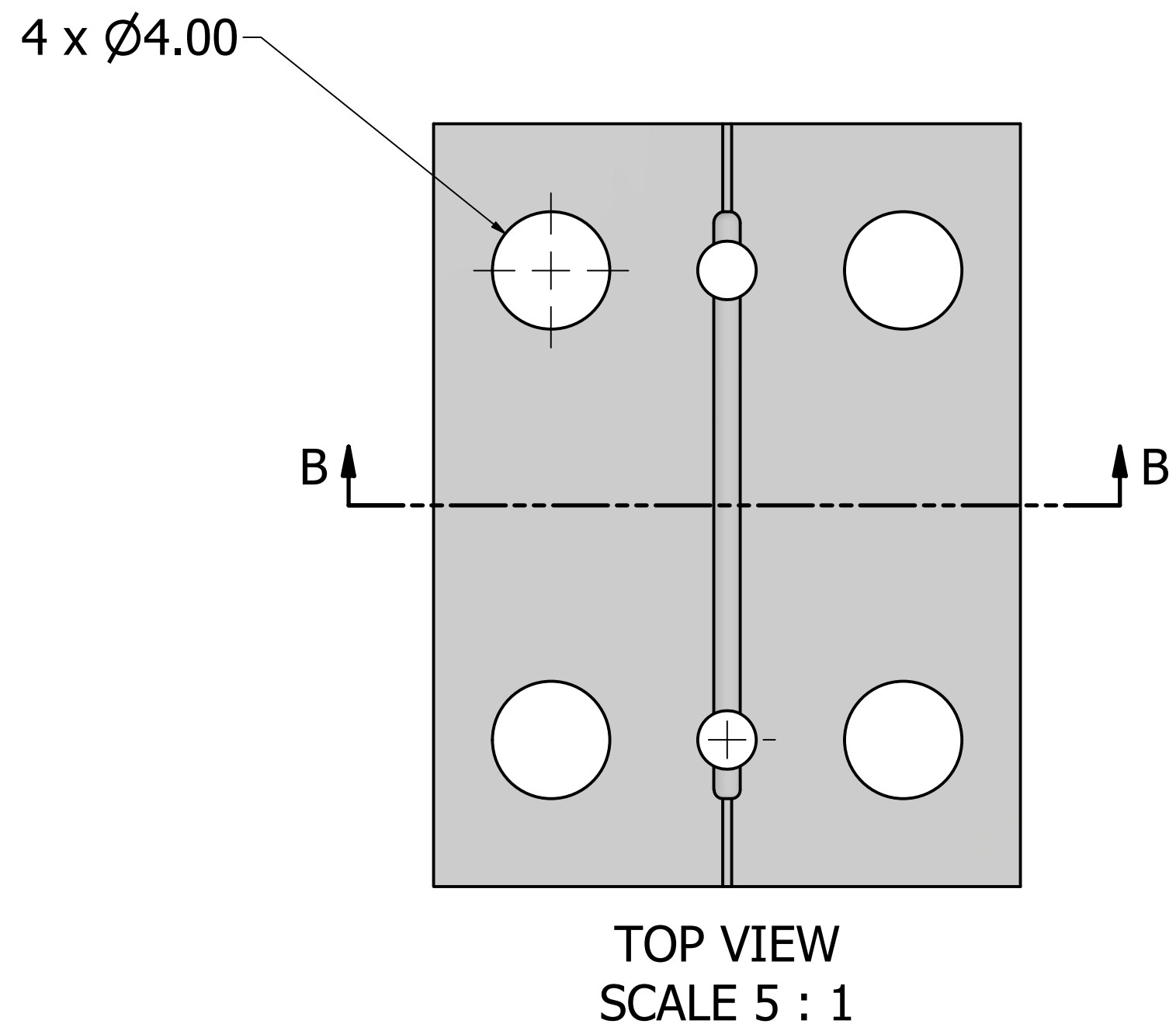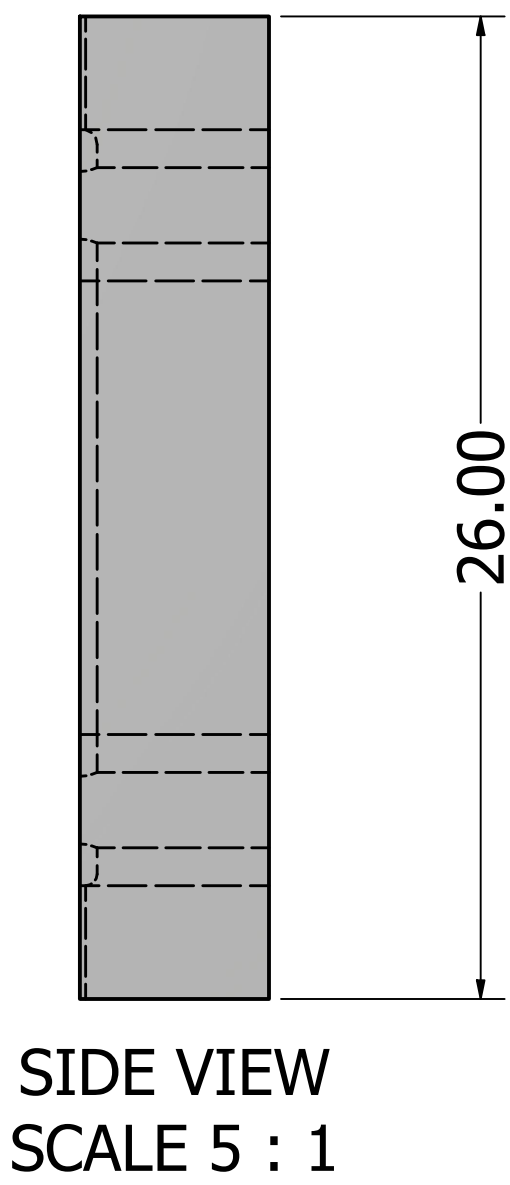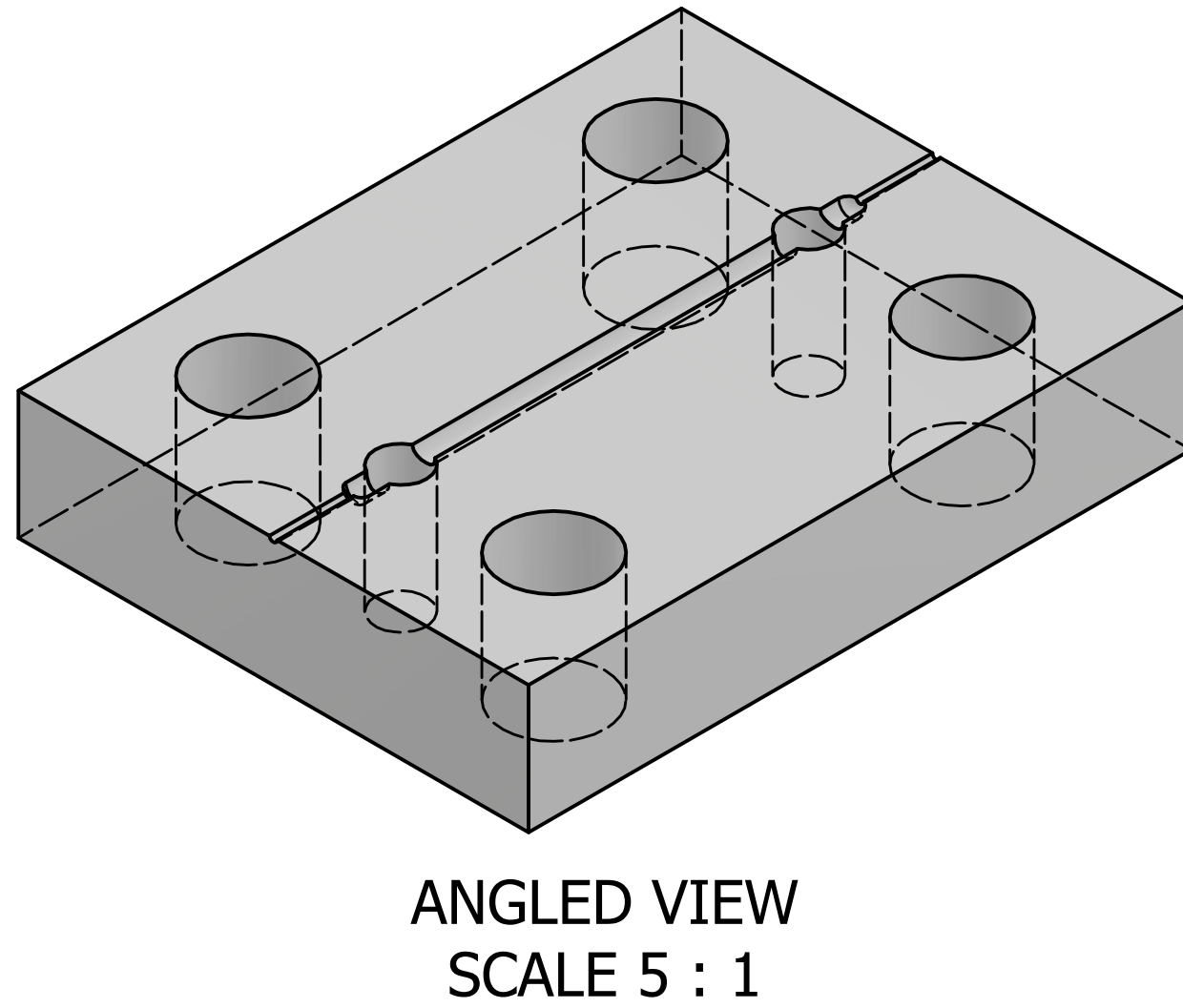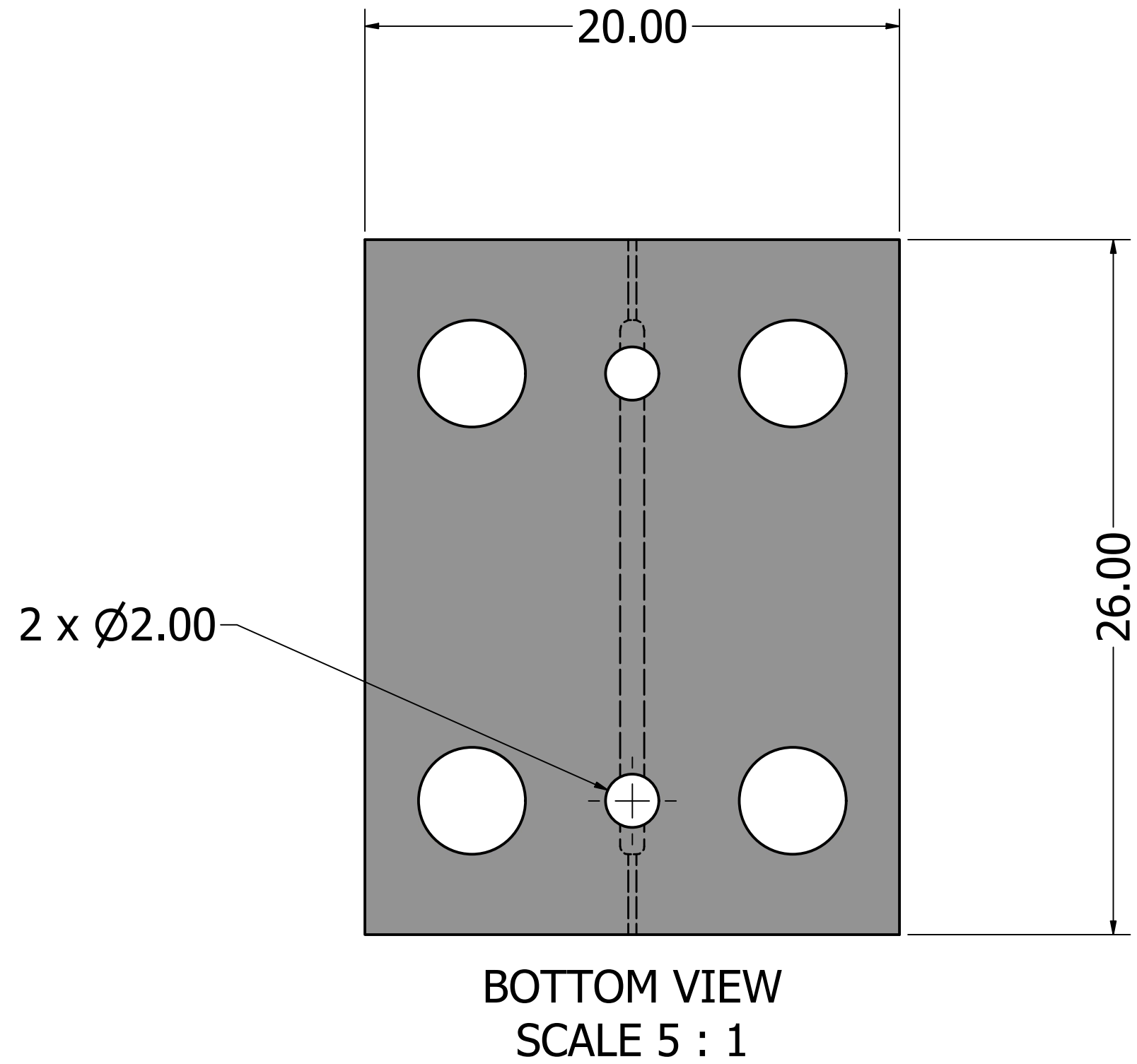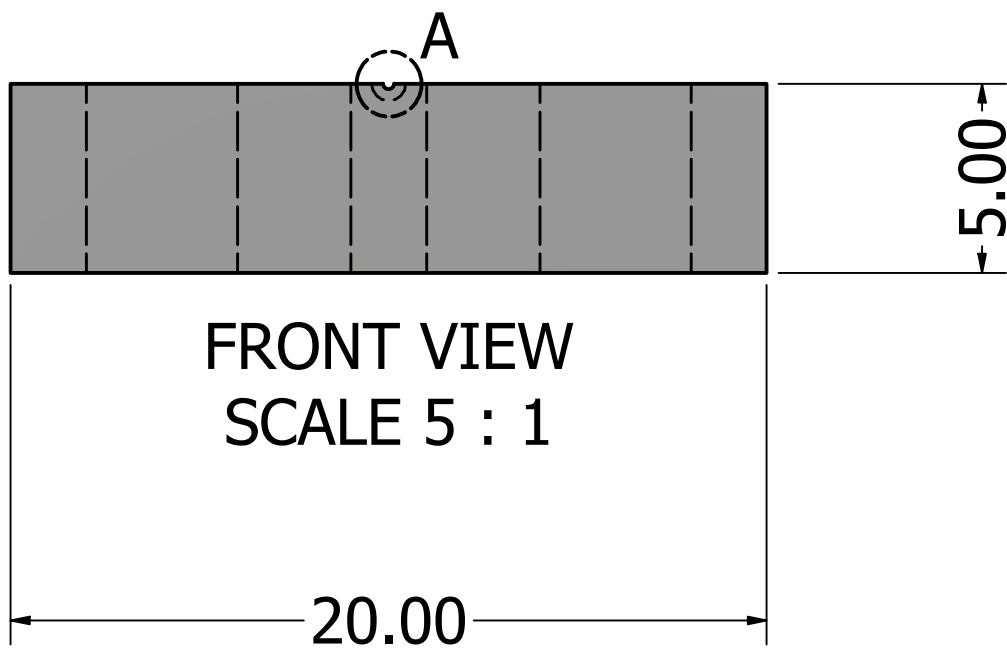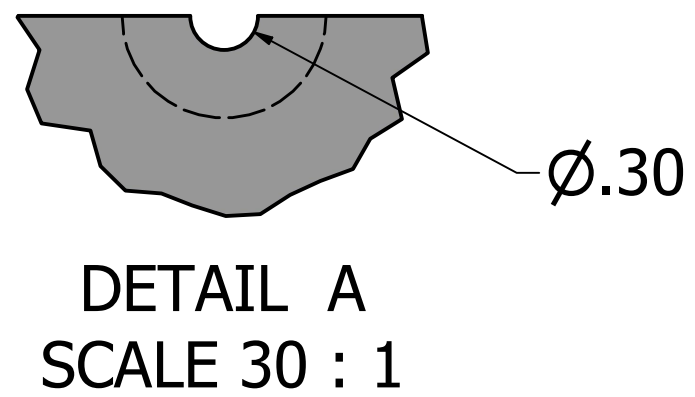

Francisco Zurita  
02/07/2022  
All units in mm

Supplement: Supplementary file 10 — Supplementary Information 8. [file 41598_2022_13679_MOESM10_ESM.pdf]
